# European Society of Cardiology quality indicators for the management of patients with ventricular arrhythmias and the prevention of sudden cardiac death: Developed in collaboration with the European Heart Rhythm Association of the European Society of Cardiology

**DOI:** 10.1093/europace/euac114

**Published:** 2022-08-26

**Authors:** Suleman Aktaa, Stylianos Tzeis, Chris P Gale, Michael J Ackerman, Elena Arbelo, Elijah R Behr, Lia Crotti, Andre d'Avila, Christian de Chillou, Thomas Deneke, Márcio Figueiredo, Tim Friede, Christophe Leclercq, Jose L Merino, Chris Semsarian, Axel Verstrael, Katja Zeppenfeld, Jacob Tfelt-Hansen, Tobias Reichlin

**Affiliations:** Leeds Institute for Data Analytics, University of Leeds, Leeds LS29JT, UK; Leeds Institute of Cardiovascular and Metabolic Medicine, University of Leeds, Leeds LS29JT, UK; Department of Cardiology, Leeds Teaching Hospitals NHS Trust, Leeds LS1 3EX, UK; Mitera Hospital, Hygeia Group, Athens 15123, Greece; Leeds Institute for Data Analytics, University of Leeds, Leeds LS29JT, UK; Leeds Institute of Cardiovascular and Metabolic Medicine, University of Leeds, Leeds LS29JT, UK; Department of Cardiology, Leeds Teaching Hospitals NHS Trust, Leeds LS1 3EX, UK; Departments of Cardiovascular Medicine, Pediatric and Adolescent Medicine, and Molecular Pharmacology & Experimental Therapeutics, Divisions of Heart Rhythm Services and Pediatric Cardiology, Windland Smith Rice Genetic Heart Rhythm Clinic and Windland Smith Rice Sudden Death Genomics Laboratory, Mayo Clinic, Rochester, MN 55905, USA; Arrhythmia Section, Cardiology Department, Hospital Clínic, Universitat de Barcelona, Barcelona 08007, Spain; IDIBAPS, Institut d’Investigació August Pi i Sunyer (IDIBAPS), Barcelona 08036, Spain; Centro de Investigación Biomédica en Red de Enfermedades Cardiovasculares (CIBERCV), Madrid 28029, Spain; Cardiovascular Clinical Academic Group and Cardiology Research Centre, St. George’s, University of London, London SW17 0RE, UK; St. George’s University Hospitals NHS Foundation Trust, London SW17 0QT, UK; Department of Cardiology, Istituto Auxologico Italiano, IRCCS, Milan 20149, Italy; Departments of Medicine and Surgery, University of Milano-Bicocca, Milan 20126, Italy; Director – Cardiac Arrhythmia Service The Harvard Thorndike EP Institute Beth Israel Deaconess Medical Center Harvard Medical School, Boston, MA 02215, USA; Department of Cardiology, University Hospital Nancy, Vandœuvre lès Nancy 54500, France; Heart Center Rhön-Clinic Bad Neustadt, Clinic for Interventional Electrophysiology, Bad Neustadt 97616, Germany; Cardiology, Electrophysiology Service, University of Campinas (UNICAMP) Hospital, Campinas 13083-888, Brazil; Department of Medical Statistics, University Medical Center Göttingen, Göttingen, Germany; and DZHK (German Centre for Cardiovascular Research), partner site Göttingen, Göttingen 10785, Germany; University of Rennes, CHU Rennes, LTSI-UMR1099, Rennes 35042, France; La Paz University Hospital, IdiPaz, Autonoma University, Madrid 28046, Spain; Agnes Ginges Centre for Molecular Cardiology at Centenary Institute, University of Sydney, Sydney 2050, Australia; Faculty of Medicine and Health, University of Sydney, Sydney 2050, Australia; Department of Cardiology, Royal Prince Alfred Hospital, Sydney 2050, Australia; Patient representative, the ESC Patient Forum, Belgium; Department of Cardiology, Leiden University Medical Center, Albinusdreef 2, ZA Leiden 2333, The Netherlands; Section of genetics, Department of Forensic Medicine, Faculty of Medical Sciences, University of Copenhagen, Copenhagen 2100, Denmark; The Department of Cardiology, The Heart Centre, Copenhagen University Hospital, Rigshospitalet 2100, Denmark; Department of Cardiology, Inselspial Bern, Bern University Hospital, University of Bern, Bern 3010, Switzerland

**Keywords:** Ventricular arrhythmias, Sudden cardiac death, Quality indicators, Treatment, Accountability, Clinical practice guidelines, Outcomes

## Abstract

To develop a suite of quality indicators (QIs) for the management of patients with ventricular arrhythmias (VA) and the prevention of sudden cardiac death (SCD). The Working Group comprised experts in heart rhythm management including Task Force members of the 2022 European Society of Cardiology (ESC) Clinical Practice Guidelines for the management of patients with VA and the prevention of SCD, members of the European Heart Rhythm Association, international experts, and a patient representative. We followed the ESC methodology for QI development, which involves (i) the identification of the key domains of care for the management of patients with VA and the prevention of SCD by constructing a conceptual framework of care, (ii) the development of candidate QIs by conducting a systematic review of the literature, (iii) the selection of the final set of QIs using a modified-Delphi method, and (iv) the evaluation of the feasibility of the developed QIs. We identified eight domains of care for the management of patients with VA and the prevention of SCD: (i) structural framework, (ii) screening and diagnosis, (iii) risk stratification, (iv) patient education and lifestyle modification, (v) pharmacological treatment, (vi) device therapy, (vii) catheter ablation, and (viii) outcomes, which included 17 main and 4 secondary QIs across these domains. Following a standardized methodology, we developed 21 QIs for the management of patients with VA and the prevention of SCD. The implementation of these QIs will improve the care and outcomes of patients with VA and contribute to the prevention of SCD.

**What’s new?**

- Quality indicators have been constructed for the management of ventricular arrhythmias and the prevention of sudden cardiac death using the European Society of Cardiology (ESC) methodology for quality indicator development and in collaboration with the European Heart Rhythm Association.
- These quality indicators are aligned with the 2022 ESC guidelines for the management of patients with ventricular arrhythmias and the prevention of sudden cardiac death.
- In total, 17 main and 4 secondary quality indicators have been selected across 8 domains of care: (i) Structural framework, (ii) screening and diagnosis, (iii) risk stratification, (iv) patient education and lifestyle modification, (v) pharmacological treatment, (vi) device therapy, (vii) catheter ablation, and (viii) outcomes.

## Introduction

Sudden cardiac death (SCD) remains a major healthcare challenge accounting for 10–15% of all deaths in Europe.^1,2^ Moreover, evidence suggests variation in the implementation of SCD preventive measures within and between countries.^3–8^ This variation calls for the development of new initiatives which may help identify areas for quality improvement in the management of patients with ventricular arrhythmias (VA) and for the prevention of premature deaths.

Quality indicators (QIs) are tools that may be used to measure adherence to and the outcomes from the uptake of guideline-recommended therapies.^9^ Given that QIs relate to discrete aspects of care, the use of QIs allows more informed interpretation of ‘real-world’ data to help address the ‘second translational gap’.^10^ As such, the European Society of Cardiology (ESC) has established suites of QIs for people with and at risk of cardiovascular disease, but until now has not developed QIs for the management of VA and the prevention of SCD.^11–15^ Although performance and quality measures exist for SCD,^16^ they predate the current clinical practice guidelines.

In parallel to the writing of the 2022 ESC Clinical Practice Guidelines for the management of patients with VA and the prevention of SCD and in collaboration with the European Heart Rhythm Association (EHRA) of the ESC, the QI Working Group for VA and SCD prevention was established to develop the first set of QIs by the ESC for this group of patients. By producing a suite of QIs which align with the current recommendations for the management of patients with VA and the prevention of SCD, it is anticipated that standardized evaluation of guideline adherence will be facilitated, and priority areas identified for quality improvement initiatives.

## Methods

We used the ESC methodology for the development of QIs for the quantification of cardiovascular care and outcomes.^9^ This methodology comprises: (i) the identification of key domains of care for the management of VA and the prevention of SCD by constructing a conceptual framework of care, (ii) the development of candidate QIs by conducting a systematic review of the literature, (iii) the selection of the final set of QIs using a modified-Delphi method, and (iv) the evaluation of the feasibility of the developed QIs.^9^

The developed QIs were classified as structural, process or outcome indicators.^9^ Structural QIs assess quality of care at the organizational level, process QIs evaluate quality of care at the level of the patient, and outcome QIs capture the outcomes of care delivery. The ESC QIs are categorized as main and secondary indicators with main QIs scoring higher for validity and feasibility.^9^

### Members of the working group

The international Working Group was formed in April 2021 and comprised healthcare professionals with expertise in the management of patients with VA and the prevention of SCD, Task Force members of the respective ESC Clinical Practice Guidelines,^17^ members of EHRA, members of the ESC QI Committee and a patient representative.

### Domains of care

**Figure 1:**
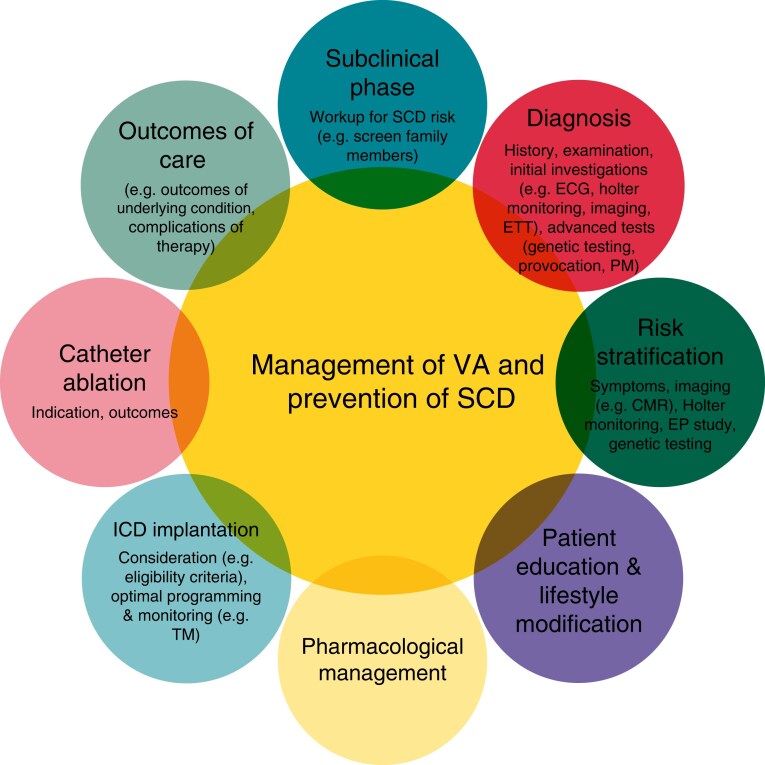
Conceptual framework for the management of patients with ventricular arrhythmia and sudden cardiac death prevention. CMR = cardiac magnetic resonance, ETT = exercise tolerance test, EP = electrophysiology, ICD = implantable cardioverter-defibrillator, PM = post-mortem, SCD = sudden cardiac death, TM = telemonitoring, VA = ventricular arrhythmias.

Following the formation of the Working Group, the members defined the target population for whom the QIs are applicable as SCD victims, survivors of SCA, and patients with VA or other conditions that are associated with SCD (e.g. primary electrical diseases, inherited disorders, and heart failure with reduced ejection fraction). The Working Group also identified the key domains of the care for the target population by conceptually illustrating the patient journey during the care delivery process (*Figure 1*).^9^

For the process QIs, the Working Group defined the patients who are eligible for the measured care process (denominator), the accomplishment criteria for the QI (numerator), and the time point at which the assessment is performed (measurement period). For the structural QIs, only numerator definitions were provided given these are binary measurements (yes, no) which capture information about the availability of resources and infrastructure.^9^

### Systematic review

#### Search strategy

A systematic review of the published literature was conducted in accordance with the Preferred Reporting Items for Systematic Review and Meta-analyses statement (see Supplementary material online, *Appendix Table A1*).^18^ We searched two online bibliographic databases, MEDLINE and Embase via OVID®. The initial search strategy was developed in MEDLINE using keywords with a variety of medical subject headings (MeSH) terms (see Supplementary material online, *Appendix Table A2*).

We included randomized controlled and observational studies, including publications from clinical registries. We included the main publications of the major trials and registries from which our search obtained only sub-studies and reviewed the studies included in the retrieved systematic reviews and meta-analyses against our inclusion criteria. The search was restricted to English language and publication dates between 01 January 2015 and 15 June 2021 given the year 2015 corresponds to the publication of the ESC Clinical Practice Guidelines for VA and SCD.^2^

#### Eligibility criteria

We included articles fulfilling the following criteria: (i) the study population was adults (age ≥18 years) with a prior history, family history or an established risk for SCA, (ii) the study defined an intervention (structural or process aspect of care) for which at least one outcome measure was evaluated, (iii) the outcome measures were hard endpoints (e.g. mortality, re-admission) or patient reported outcomes (e.g. quality of life), (iv) the study provided definitions for the intervention and outcome measure(s) evaluated, and (v) the study was a peer-reviewed randomized controlled trial or observational study.

#### Study selection

EndNote X9 was used for reference management and for duplicate removal. Three reviewers (S.A., T.R., and S.T.) independently examined the abstracts of the studies retrieved from the search against the inclusion criteria. Disagreements were resolved through discussion and a full text review of the debated article.

#### Quality assessment and data extraction

Studies that met the eligibility criteria were included in the initial phase of the review. A broad inclusion was used to ensure that the list of initial (candidate) QIs encompassed the range of care delivery. The full texts of the included articles were reviewed by three authors (S.A., T.R., and S.T.) and for each study both the intervention(s), and the outcome measure(s) evaluated were extracted to an Excel spreadsheet. Definitions of the extracted data items were obtained when provided in the study.

#### Clinical Practice Guidelines, consensus documents, and QIs

Existing QIs, consensus documents, and Clinical Practice Guidelines pertinent to the management of VA and the prevention of SCD were reviewed.^11–16^ The Working Group opted not to replicate aspects of care described in previous ESC QI suites. As such, the present document is complementary to published ESC QI documents.

The goal of the Clinical Practice Guidelines review was to assess the suitability of their recommendations with the strongest association with benefit and harm (Class I and III, respectively) against the ESC criteria for QIs (see Supplementary material online, *Appendix Table A3*).^9^

### Data synthesis

#### Modified Delphi process

We used the modified Delphi method to evaluate the candidate QIs that were derived from the literature.^9^ The ESC criteria for QI development (see Supplementary material online, *Appendix Table A3*) were shared with the Working Group members prior to the voting in order to guide the selection process. Candidate QIs were graded according to a nine-point ordinal scale for both validity and feasibility by each Working Group member using an on-line questionnaire.^9^ Two rounds in total were conducted, with a number of teleconferences after each round to discuss the results of the vote and address any concerns or ambiguities.

#### Analysing voting results

Ratings of 1 to 3 were defined as meaning that the QI was not valid/feasible; ratings 4 to 6 that the QI was of an uncertain validity/feasibility; and ratings of 7 to 9 that the QI was valid/feasible. For each candidate QI, the median and the mean deviation from the median were calculated to evaluate the central tendency and the dispersion of the votes. Indicators with median scores ≥7 for validity, ≥4 for feasibility, and with minimal dispersion were included in the final set of QIs.^9^ Those QIs meeting the inclusion criteria in the first voting round formed the main QIs and those that met the inclusion criteria after a second round of voting formed the secondary QIs.

## Results

### Domains of care

**Figure 2:**
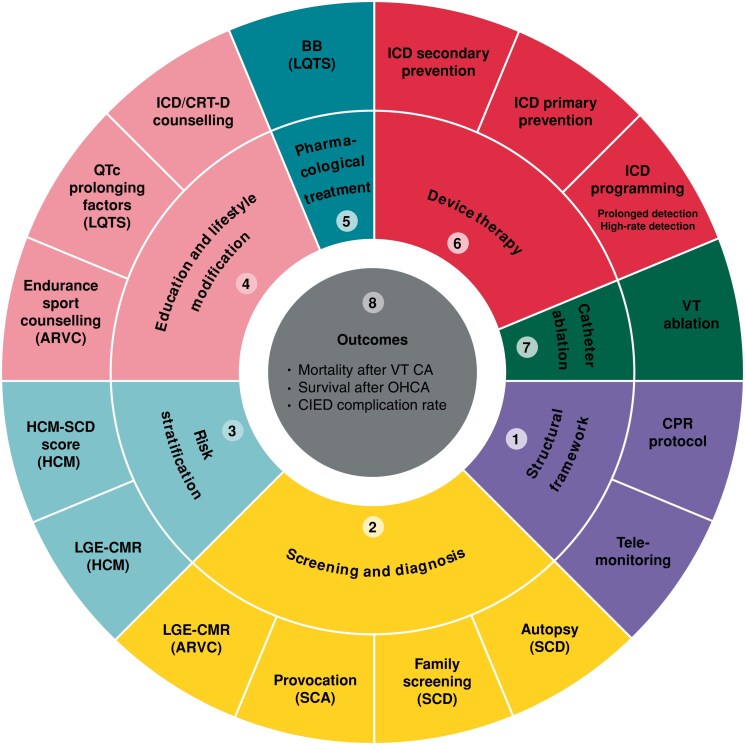
2022 ESC EHRA QIs for VA and SCD prevention. ARVC = arrhythmogenic right ventricular cardiomyopathy, BB = beta-blockers, CMR = cardiac magnetic resonance, CA = catheter ablation, CIED = cardiac implantable electronic devices, CPR = cardiopulmonary resuscitation, CRT-D = cardiac resynchronization therapy-defibrillator, DC = discharge, ESC = European Society of Cardiology, HCM = hypertrophic cardiomyopathy, ICD = implantable cardioverter-defibrillator, LGE = late gadolinium enhancement, LQTS = long QT syndrome, OHCA = out-of-hospital cardiac arrest, SCD = sudden cardiac death, VA = ventricular arrhythmia, VT = ventricular tachycardia.

In total, eight domains of care for the management of patients with VA and the prevention of SCD were identified by the Working Group. These domains included: (i) structural framework, (ii) screening and diagnosis, (iii) risk stratification, (iv) patient education and lifestyle modification, (v) pharmacological treatment, (vi) device therapy, (vii) catheter ablation, and (viii) outcomes (*Figure 2*).

### Quality indicators

#### Systematic review results

**Figure 3:**
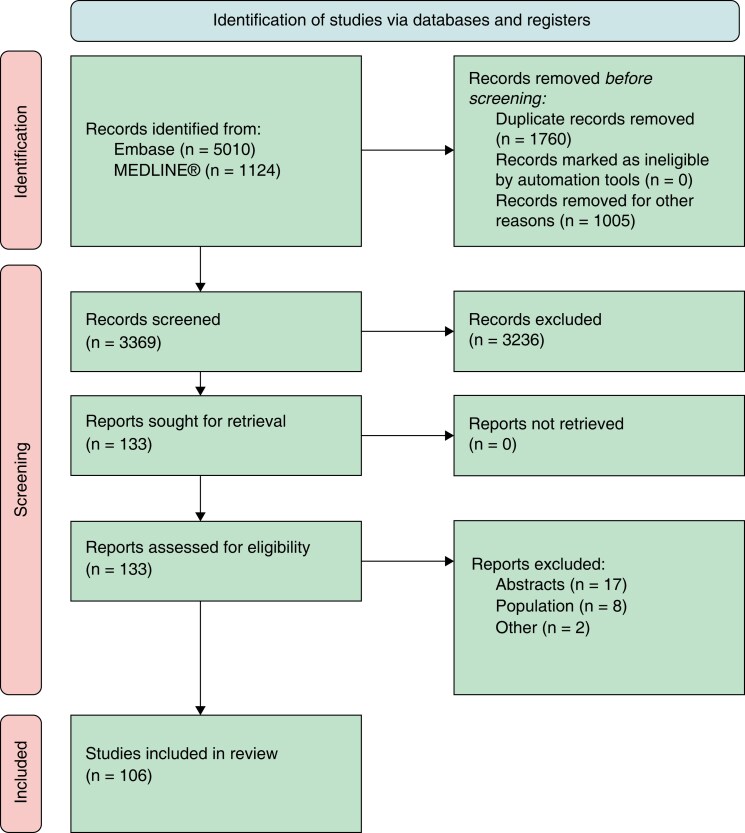
Preferred reporting items for systematic reviews and meta-analyses flowchart for the systematic review.

The literature search retrieved 3,369 articles, of which 107 met the inclusion criteria (*Figure 3*) and were used to extract 75 candidate QIs for the first Delphi round. Of those, 25 (33%) met the criteria for inclusion as main QIs, 39 (52%) were excluded and 11 (15%) QIs were deemed inconclusive. Following Working Group membership discussion, 8 (32%) of the main QIs were downgraded and subsequently reconsidered in a second Delphi round alongside the inconclusive ones. Thus, a total of 19 QIs were included in the second Delphi round, after which 4 (21%) QIs met the inclusion criteria and were selected as secondary QIs (*Figure 2*). As such, a total of 17 main and 4 secondary QIs were included in the final set of the 2022 ESC QIs for the management of VA and the prevention of SCD (*Table 1*).

**Table 1:** List of ESC QIs for the management of VA and the prevention of SCD

**Domain 1. Structural QIs**	
**QI 01M01:** Healthcare centres with inpatient service, which have a dedicated team to deliver cardiopulmonary resuscitation (CPR) 24/7 with a written CPR protocol.	
Numerator	Healthcare centres with dedicated team to deliver CPR 24/7 with a written CPR protocol.
**QI 01M02:** Healthcare centres involved in the management of SCA survivors and those at risk of SCD that have available protocols for the implementation & surveillance of remote monitoring for patients with CIED.	
Numerator	Healthcare centres with available protocols for the implementation & surveillance of remote monitoring for patients with CIED.
**Domain 2. Screening and diagnosis**	
**QI 02M01**: Proportion of young (<50 years) unexplained SCD victims who are referred for comprehensive autopsy including cardiac histopathology, post-mortem genetic testing, and, where indicated, toxicology.	
Numerator	Number of young (<50 years) unexplained SCD victims who are referred for comprehensive autopsy including cardiac histopathology, post-mortem genetic testing and, where indicated, toxicology.
Denominator	Number of young (<50 years) unexplained SCD victims.
**QI 02M02:** Proportion of SCD victims with likely heritable phenotype whose families receive a clinical and/or genetic workup for inherited cardiovascular conditions.	
Numerator	Number of SCD victims with likely heritable phenotype whose families receive a clinical and/or genetic workup for inherited cardiovascular conditions.
Denominator	Number of SCD victims with likely heritable phenotype.
**QI 02M03:** Proportion of unexplained SCA survivors who undergo pharmacological provocation testing.	
Numerator	Number of unexplained SCA survivors who undergo pharmacological provocation testing.
Denominator	Number of unexplained SCA survivors.
**QI 02M04:** Proportion of patients with arrhythmogenic right ventricular cardiomyopathy who undergo LGE-CMR at the time of diagnosis.	
Numerator	Number of patients with arrhythmogenic right ventricular cardiomyopathy who undergo LGE-CMR at the time of diagnosis.
Denominator	Number of patients with arrhythmogenic right ventricular cardiomyopathy.
**Domain 3. Risk stratification**	
**QI 03M01:** Proportion of patients with hypertrophic cardiomyopathy (HCM) who undergo an assessment of their risk of SCD using the HCM SCD risk score at the time of initial evaluation.	
Numerator	Number of patients with hypertrophic cardiomyopathy who undergo an assessment of their risk of SCD using the HCM SCD risk score at the time of initial evaluation.
Denominator	Number of patients with hypertrophic cardiomyopathy.
**QI 03M02**: Proportion of patients with hypertrophic cardiomyopathy who undergo LGE-CMR at the time of initial evaluation.	
Numerator	Number of patients with hypertrophic cardiomyopathy who undergo LGE-CMR at the time of initial evaluation.
Denominator	Number of patients with hypertrophic cardiomyopathy.
**Domain 4. Patient education and lifestyle modifications**	
**QI 04M01:** Proportion of patients with arrhythmogenic cardiomyopathy who receive counselling about avoidance of high intensity and endurance sports.	
Numerator	Number of patients with arrhythmogenic cardiomyopathy who receive counselling about avoidance of high intensity and endurance sports.
Denominator	Number of patients with arrhythmogenic cardiomyopathy.
**QI 04M02:** Proportion of patients with congenital LQTS who receive counselling about all the following: the risk of electrolyte abnormalities (e.g. diarrhoea and vomiting, but also use of diuretics), the avoidance of QT-prolonging drugs (www.crediblemeds.org), and the avoidance of genotype-specific triggers for arrhythmias.	
Numerator	Number of patients with congenital LQTS who receive counselling about all the following: the risk of electrolyte abnormalities (e.g. diarrhoea & vomiting, but also use of diuretics), the avoidance of QT-prolonging drugs, and the avoidance of genotype-specific triggers for arrhythmias.
Denominator	Number of patients with congenital LQTS.
**QI 04S01:** Proportion of patients with an ICD/CRT-D who receive counselling about living with a defibrillator.	
Numerator	Number of patients with an ICD/CRT-D who receive counselling about living with a defibrillator.
Denominator	Number of patients with an ICD/CRT-D.
**Domain 5. Pharmacological treatment**	
**QI 05M01:** Proportion of patients with congenital LQTS who receive beta-blockers.	
Numerator	Number of patients with congenital LQTS who receive beta-blockers.
Denominator	Number of patients with congenital LQTS.
**Domain 6. Device therapy**	
**QI 06M01:** Proportion of VT/VF cardiac arrest survivors (or those with spontaneous sustained VT causing syncope or haemodynamic instability) without a reversible cause who have a life expectancy >1 year and receive secondary prevention ICD implantation.	
Numerator	Number of VT/VF cardiac arrest survivors (or those with spontaneous sustained VT causing syncope or haemodynamic instability) without a reversible cause who have a life expectancy >1 year and receive secondary prevention ICD implantation.
Denominator	Number of VT/VF cardiac arrest survivors (or those with spontaneous sustained VT causing syncope or haemodynamic instability) without a reversible cause who have a life expectancy >1 year.
**QI 06M02:** Proportion of patients with ischaemic cardiomyopathy, NYHA class II-III who have EF≤35% despite ≥3 months of OMT and life expectancy > 1 year who receive ICD for primary prevention of SCD.	
Numerator	Number of patients with ischaemic cardiomyopathy, NYHA class II-III who have EF≤35% despite ≥3 months of OMT and life expectancy > 1 year who receive ICD for primary prevention of SCD.
Denominator	Number of patients with ischaemic cardiomyopathy, NYHA class II-III who have EF≤35% despite ≥3 months of OMT and life expectancy > 1 year.
**QI 06M03:** Proportion of patients with primary prevention ICD whose device is programmed to a prolonged detection strategy and/or high-rate programming strategy.	
Numerator	Number of patients with primary prevention ICD whose device is programmed to a prolonged detection strategy and/or high-rate programming strategy.
Denominator	Number of patients with primary prevention ICD.
**Domain 7. Catheter ablation**	
**QI 07M01:** Proportion of patients with ischaemic cardiomyopathy and recurrent, symptomatic sustained monomorphic VT despite chronic amiodarone therapy who receive VT ablation.	
Numerator	Number of patients with ischaemic cardiomyopathy and recurrent, symptomatic sustained monomorphic VT despite chronic amiodarone therapy who receive VT ablation.
Denominator	Number of patients with ischaemic cardiomyopathy and recurrent, symptomatic sustained monomorphic VT despite chronic amiodarone therapy.
**Domain 8. Outcomes**	
**QI 08M01:** All-cause mortality at 30 days following VT ablation.	
Numerator	Number of patients who died from any cause within 30 days following VT ablation
Denominator	Number of patients who underwent VT ablation.
**QI 08M02:** Survival to hospital discharge after cardiac arrest	
Numerator	Number of patients who survive to hospital discharge after cardiac arrest
Denominator	Number of patients admitted with cardiac arrest
**QI 08S01:** Procedural complications 30 days following ICD implantation [ICD-related bleeding, pneumothorax, cardiac perforation, tamponade, pocket haematoma, lead displacement, infection (all requiring intervention), or death].	
Numerator	Number of patients who develop any procedural complication [ICD-related bleeding, pneumothorax, cardiac perforation, tamponade, pocket haematoma, lead displacement, infection (all requiring intervention), or death] within 30 days following ICD implantation.
Denominator	Number of patients who undergo ICD implantation.
**QI 08S02:** ICD-related infections up to 1 year following ICD implantation, replacement, or revision.	
Numerator	Number of patients who develop ICD-related infection within 1 year following ICD implantation, replacement, or revision.
Denominator	Number of patients who undergo ICD implantation, replacement or revision.
**QI 08S03:** Procedural complications 30 days following VT-ablation (vascular complications, tamponade, stroke, complete heart block).	
Numerator	Number of patients who develop any procedural complication (vascular complications, tamponade, stroke, complete heart block) within 30 days following VT-ablation.
Denominator	Number of patients who undergo VT-ablation.

CIED = cardiac implantable electronic devices; CRT = cardiac resynchronization therapy; ESC = European Society of Cardiology; ICD = implantable cardioverter defibrillator; LGE-CMR = late gadolinium enhancement-cardiac magnetic resonance; LQTS = long-QT syndrome; NYHA = New York Heart Association; OMT = optimal medical therapy; QIs = quality indicators; SCD = sudden cardiac death; SCA = sudden cardiac arrest; VA = ventricular arrhythmias; VT = ventricular tachycardia; VF = ventricular fibrillation.

### Domain 1: structural framework

Organizational components in healthcare centres are important for optimizing the management of patients with VA and those at risk for SCD.^19^ Such structural measures are relevant to the standards of care at the institutional level which may impact patient outcomes.^19^ In this context, the availability of a dedicated and competent cardiac arrest team that delivers a prompt and high-quality cardiopulmonary resuscitation according to the European Resuscitation Guidelines is an indicator of care quality for SCD prevention **(QI 01M01)**.^20^

The follow-up of patients with cardiac implantable electronic devices (CIED) is an important aspect of care delivery for patients with VA and those at risk of SCD. Remote CIED monitoring has been demonstrated to prevent inappropriate defibrillator shocks and to improve clinical outcomes and thus is a QI of CIED follow up **(QI 01M02)**.^21,22^

### Domain 2: screening and diagnosis

Identifying the underlying aetiology for cardiac arrhythmias is the primary goal not only for preventing further episodes in aborted SCD victims, but also for guiding familial investigation in case of a documented or suspected inherited cardiac disease.

The performance of an autopsy for SCD is necessary for the investigation of potential inherited cardiac diseases, particularly in unexplained SCD in young (age < 50 years) individuals. As such, the performance of a comprehensive autopsy including cardiac histopathology and post-mortem genetic testing (also known as the molecular autopsy) targeted to not only primary electrical diseases but also concealed cardiomyopathies, with/without toxicology assessment (e.g. polypharmacy or drug abuse) in this group of patients is an indicator of care quality (**QI 02M01**).^23^

Screening the relatives of those with SCD is recommended to identify asymptomatic individuals at potential risk of lethal arrhythmias due to an inherited cardiac disease.^24,25^ Having a standardized protocol for such a screening is an indicator of SCD prevention care quality (**QI 02M02**).

In patients with unexplained SCA, pharmacological provocation testing increases the diagnostic yield and is an indicator of care quality (**QI 02M03**).^26^

Advanced imaging modalities such as late gadolinium enhancement (LGE) on cardiac magnetic resonance imaging (cMRI) play a major role in the diagnosis of arrhythmogenic right ventricular cardiomyopathy (ARVC) (**QI 02M04**).^17^

### Domain 3: risk stratification

Risk assessment may identify individuals at higher risk of VA or SCD and helps determine risk-mitigation strategies, such as pharmacological therapy or implantable cardioverter defibrillator (ICD) implantation.^17^

For patients with hypertrophic cardiomyopathy (HCM), the HCM-SCD risk score provides an estimate of 5-year risk of SCD for patients with HCM.^27^ This algorithm has been internally and externally validated and improves SCD risk prediction when compared with other prediction models.^27,28^ Patients with a predicted 5-year risk of SCD ≥ 6% have the highest event rate and the most favourable risk-benefit ratio for ICD implantation. The use of the HCM-SCD risk score therefore forms a QI for the prevention of SCD in patients with HCM (**QI 03M01**).

In addition, LGE-CMR helps identify the presence of fibrosis in patients with HCM and has prognostic implications. Thus, LGE-CMR at the time of initial evaluation has been selected as an indicator of care quality for this group of patients (**QI 03M02**).^17^

### Domain 4: patient education and lifestyle modifications

Lifestyle habits and physical factors may induce VA in patients with certain types of underlying heart disease.^29–31^ Patient education is recommended to reduce the risk of VA and SCD. Whilst adopting a ‘healthy’ lifestyle including smoking cessation, regular exercise, healthy diet, and weight loss reduces the risk of SCD,^32^ specific lifestyle modifications may be needed for certain underlying arrhythmogenic disorders.

ARVC is an inherited disease whose progression and clinical course, including VA occurrence, is adversely affected by high-intensity exercise.^29–31,33^ Thus, patient counselling on avoidance of vigorous exercise is an essential component of SCD prevention in this group of patients (**QI 04M01**).

For patients with long QT syndrome (LQTS), several triggers have been identified for different types of the disorder. As such, educating patients on the avoidance of those triggers is of paramount importance to reduce the risk of SCD in patients with LQTS. Furthermore, education is essential to reduce modifiable factors, such as QT prolonging medications (www.crediblemeds.org) and electrolyte abnormalities (**QI 04M02**).^34^

An ICD/cardiac resynchronization therapy-defibrillator (CRT-D) can affect daily life and mental health.^35,36^ Having an ICD also incurs sensitive discussions about device deactivation among patients and families.^37^ Accordingly, it is recommended that patients with an ICD/CRT-D receive counselling about living with an ICD (**QI 04S01**).

### Domain 5: pharmacological treatment

Adrenergic activation is a well-documented trigger of VA in patients with congenital LQTS. Beta blockers reduce the burden of syncope and SCD in patients with LQTS.^38–40^ Non-selective beta blockers propranolol and nadolol are even more protective against breakthrough arrhythmic events in LQTS patients.^41^ Thus, beta blockers constitute the mainstay of the management of patients with congenital LQTS. Whilst certain types of LQTS may have greater benefit from beta blocker treatment compared with other types,^42^ improved outcomes are observed across the whole spectrum of LQTS and is thus an indicator of care quality in this group of patients (**QI 05M01**).^43^

### Domain 6: device therapy

ICD therapy is considered a primary therapeutic option for the prevention of arrhythmic death. Evidence supports the use of ICD for secondary and primary prevention of SCD in eligible patients.^44–49^ For secondary prevention after cardiac arrest or sustained symptomatic ventricular tachycardia (haemodynamically not tolerated), where no reversible cause is identified, ICD implantation reduces all-cause mortality when compared with medical treatment and is thus a QI for SCD prevention (**QI 06M01**).

For the primary prevention of SCD, the strongest evidence is in favour of device therapy in patients with symptomatic heart failure and a left ventricular ejection fraction ≤ 35% despite ≥ 3 months of optimal medical therapy.^50^ For those with non-ischaemic heart failure, data supporting the benefit derived from primary prevention ICD implantation is less robust.^51^ Therefore, the working group voted in favour of adopting the proportion of ischaemic cardiomyopathy patients, New York Heart Association class II-III who have a left ventricular ejection fraction ≤35% and ≥ 3 months of optimal medical therapy and a life expectancy > 1 year who receive ICD for primary prevention of SCD as a QI of appropriate device therapy (**QI 06M02**).

Customization of optimal ICD settings is associated with a reduced number of ICD therapies and improved patient outcome.^52,53^ Programming of prolonged tachyarrhythmia detection settings and high-rate tachycardia detection thresholds is effective in reducing the overall therapy burden, without impairing patient safety among primary prevention ICD recipients.^54–56^ Accordingly, detailed programming recommendations are now available in expert consensus papers.^57,58^ The proportion of primary prevention ICD recipients whose device is programmed to a prolonged detection strategy and/or high-rate programming strategy is proposed as an indicator of high-quality care (**QI 06M03**).

### Domain 7: catheter ablation

Despite the efficacy of ICD therapy in terminating VT episodes, the burden of ICD interventions should be minimized because ICD shocks are associated with poorer quality of life and outcomes.^59,60^ Catheter ablation is an effective intervention in reducing VT recurrences in specific types of VTs with subsequent improvement in survival.^61^ Treatment alternatives in ICD recipients experiencing VT recurrences despite antiarrhythmic drug treatment would be either escalation of antiarrhythmic drug or catheter ablation. VT ablation is more effective in reducing recurrent VT episodes and appropriate ICD shocks than antiarrhythmic drug escalation in ischaemic cardiomyopathy with VT despite appropriate first-line antiarrhythmic drugs.^62^ Therefore, the proportion of ischaemic cardiomyopathy patients with recurrent, symptomatic sustained monomorphic VT despite chronic amiodarone therapy who receive VT ablation is a QI in provision of catheter ablation therapy (**QI 07M01**).^63,64^

### Domain 8: outcomes

Whilst VT ablation reduces ICD shocks and VT recurrence and has favourable effects on patient outcomes,^61^ it may be associated with procedural complications including stroke and death.^62^ Morbidity and mortality in the 30 days following VT ablation is not negligible.^65^ Notwithstanding that procedural complications or death within 30 days after VT ablation are not necessarily attributable to the procedure per se, but rather to the underlying heart disease or even non-cardiac causes,^62^ it remains important to monitor trends in all-cause mortality (**QI 08M01**) and procedural complications in the first 30 days following VT ablation (**QI 08S03**).

With regards to ICD procedures, complications in the first 30 days after implantation (**QI 08S01**) and procedure-related infections up to 1 year after all types of ICD implantation (**QI 08S02**) are QIs.^66^

Survival to hospital discharge after out-of-hospital cardiac arrest is determined by several factors including the organization of emergency medical service, bystander CPR-rates, post-resuscitation protocols and provision of long-term care. Survival to hospital discharge is a key indicator for monitoring changes over time within a given system and for comparison across sites (**QI 08M02**).^67^

## Discussion

This document presents the first suite of the ESC QIs for the management of patients with VA and the prevention of SCD. It was developed in collaboration with EHRA and the Task Force of the 2022 ESC guidelines for the management of patients with VA and the prevention of SCD.^17^ These 17 main and 4 secondary QIs across 8 domains of care were developed using a standardized methodology that combines evidence with expert judgment, and serve as tools to monitor and improve the management of patients with VA and to reduce the burden of SCD.

QIs have gained increased attention in recent years for two reasons. First, they provide tools for assessing, monitoring and reporting the quality of care and associated improvement initiatives within and across healthcare systems. Second, QIs support the adoption of guideline recommendations into clinical practice by translating key messages into specific and measurable QIs.^9^ This point has been recognized by the ESC and since 2020 the ESC guidelines have been accompanied by suites of QIs.^11–16^

The present document outlines key aspects for the management of VA and the prevention of SCD. The 2016 American College of Cardiology/American Heart Association (ACC/AHA) performance and quality measures for SCD prevention provided a list of important and feasible interventions, but lacks the inclusion of structural or outcome QIs which are of a particular importance in the context of VA and SCD prevention.^16^ In addition, there are no recommendations in the ACC/AHA set for the application of advanced imaging (e.g. LGE-CMR),^68^ monitoring (e.g. remote monitoring)^69^ or therapeutic (e.g. ablation)^62^ technologies for patients at risk of SCD.^16^

The QIs defined in this document may stimulate quality assessment and improvement for patients at risk of SCD, but also provide the basis for data collection across different settings. The European Unified Registries on Heart care Evaluation and Randomized Trials (EuroHeart) project,^70^ incorporates the ESC QIs for cardiovascular disease into its international registries so that standardized ‘real-world’ data and performance may be described, and care improved.

Furthermore, the QI of autopsy following a sudden unexplained death addresses the extreme heterogeneity and inequality of access across Europe. A recent survey of the EHRA Research Network and European Reference Network GUARD-Heart conducted by the Scientific Initiatives Committee and the European Cardiac Arrhythmia Genetic Focus Group of EHRA, indicated that on average, an autopsy was performed in 43% of suitable cases: 39% of respondents stated that autopsy rates were between 50% and 100%; 23% reported a rate between 25% and 49%; 31% a rate from 1% to 24%; and 7% stated that no autopsy is usually undertaken.^71^ The main reason for low autopsy rates was the lack of legal mandate which requires a Europe-wide public health initiative that this QI will measure.

The selection of the developed QIs was structured according to the ESC methodology for QI development.^9^ The conduction of a systematic review of the literature and the involvement of a far-reaching Working Group ensured that the selected set of QIs are valid measures of care quality which are also feasible and relevant to existing gaps in care delivery.

There are limitations of our work which merit consideration. The target population for these QIs was broad and included patients at risk for SCD, as well as victims of SCD and their family members. As such, the Working Group prioritized key aspects of care delivery across the whole spectrum of SCD prevention and avoided replicating relevant QIs that have recently been covered in other suites of the ESC QIs, such as these for heart failure, cardiovascular disease prevention and cardiac pacing.^11,12,14–16^ Some of the QIs relate to care that is not available in some areas of Europe (e.g. cMRI and specialist pathology). Even so, the majority of the Working Group agreed upon these measures so that they may be used in advocacy for changes in healthcare delivery. The ESC methodology used to develop the QIs relied on expert opinion, and this may have influenced the results. To minimize a bias: (i) a systematic literature review was performed as a basis for QI development; (ii) the subsequent modified Delphi method for selection of the final set of QI followed a standardized process^9^; and (iii) the members of the working group included experts in cardiac electrophysiology, cardiomyopathies, channelopathies, general cardiologist, patient representatives as well as individuals with expertise in the development of QI, and all voted independently during the Delphi process. We recommend that the QI suite is evaluated and refined as new evidence becomes available.

## Conclusions

This document defines 17 main and 4 secondary QIs across eight domains of care for the management of patients with VA and for the prevention of SCD. The QIs span the breadth of the care delivery for individuals at risk of SCD and provide a framework for quality improvement initiatives aiming to improve quality of care and outcomes for the management of VA and prevention of SCD.

## Supplementary material

Supplementary material is available at Europace online.

## Supplementary Material

euac114_Supplementary_Data

## Data Availability

The data underlying this article are available in the article and in its online supplementary material.
