# Supplementary material for: European Society of Cardiology quality indicators for the management of patients with ventricular arrhythmias and the prevention of sudden cardiac death: Developed in collaboration with the European Heart Rhythm Association of the European Society of Cardiology
Source: Europace. 2022 Aug 26;25(1):199–210. doi: 10.1093/europace/euac114 (PMC10103575; doi:10.1093/europace/euac114)
Supplement: euac114_Supplementary_Data [file euac114_supplementary_data.docx]

**Appendices**

Table A1. PRISMA checklist.

| Section/topic | # | Checklist item | Reported on page # |
| --- | --- | --- | --- |
| TITLE | | |  |
| Title | 1 | Identify the report as a systematic review, meta-analysis, or both. | 1 |
| ABSTRACT | | |  |
| Structured summary | 2 | Provide a structured summary including, as applicable: background; objectives; data sources; study eligibility criteria, participants, and interventions; study appraisal and synthesis methods; results; limitations; conclusions and implications of key findings; systematic review registration number. | 2 |
| INTRODUCTION | | |  |
| Rationale | 3 | Describe the rationale for the review in the context of what is already known. | 3 |
| Objectives | 4 | Provide an explicit statement of questions being addressed with reference to participants, interventions, comparisons, outcomes, and study design (PICOS). | 3 |
| METHODS | | |  |
| Protocol and registration | 5 | Indicate if a review protocol exists, if and where it can be accessed (e.g., Web address), and, if available, provide registration information including registration number. | N/A |
| Eligibility criteria | 6 | Specify study characteristics (e.g., PICOS, length of follow-up) and report characteristics (e.g., years considered, language, publication status) used as criteria for eligibility, giving rationale. | 5 |
| Information sources | 7 | Describe all information sources (e.g., databases with dates of coverage, contact with study authors to identify additional studies) in the search and date last searched. | 6 |
| Search | 8 | Present full electronic search strategy for at least one database, including any limits used, such that it could be repeated. | 6 |
| Study selection | 9 | State the process for selecting studies (i.e., screening, eligibility, included in systematic review, and, if applicable, included in the meta-analysis). | 6 |
| Data collection process | 10 | Describe method of data extraction from reports (e.g., piloted forms, independently, in duplicate) and any processes for obtaining and confirming data from investigators. | 6 |
| Data items | 11 | List and define all variables for which data were sought (e.g., PICOS, funding sources) and any assumptions and simplifications made. | 6 |
| Risk of bias in individual studies | 12 | Describe methods used for assessing risk of bias of individual studies (including specification of whether this was done at the study or outcome level), and how this information is to be used in any data synthesis. | N/A |
| Summary measures | 13 | State the principal summary measures (e.g., risk ratio, difference in means). | N/A |
| Synthesis of results | 14 | Describe the methods of handling data and combining results of studies, if done, including measures of consistency (e.g., I^2^) for each meta-analysis. | 7 |

Table A2. Embase and MEDLINE search terms for the systematic review.

| Database: Embase <1996 to 2021 Week 23> |
| --- |
| Search Strategy: |
| 1 exp Death, Sudden, Cardiac/ (17885) |
| 2 (sudden adj2 death).mp. or sudden death/ (75982) |
| 3 cardiac death.mp. (51377) |
| 4 ((card* or sudden) adj1 (death or arrest)).ti,ab. (129891) |
| 5 (ventric* adj1 fibrillation).ti,ab. or heart ventricle fibrillation/ or "electrical storm (heart)"/ (30199) |
| 6 or/1-5 (174452) |
| 7 heart infarction/ (222475) |
| 8 acute heart infarction/ (71988) |
| 9 acute coronary syndrome/ (62038) |
| 10 ST segment elevation/ (27044) |
| 11 non st segment elevation acute coronary syndrome/ (2046) |
| 12 infarction/ or ischemia/ or necrosis/ (150705) |
| 13 apoptosis/ and heart/ (4124) |
| 14 cell death/ and heart/ (1523) |
| 15 cell damage/ and heart/ (518) |
| 16 scar formation/ and heart/ (334) |
| 17 heart failure/ (235381) |
| 18 congestive heart failure/ (66194) |
| 19 acute heart failure/ or congestive heart failure/ (87548) |
| 20 left ventricular systolic dysfunction/ or systolic dysfunction/ or diastolic dysfunction/ or left ventricular diastolic dysfunction/ (33924) |
| 21 ischemic cardiomyopathy/ or alcoholic cardiomyopathy/ or nonischemic cardiomyopathy/ or restrictive cardiomyopathy/ or congestive cardiomyopathy/ or peripartum cardiomyopathy/ or hypertrophic cardiomyopathy/ or cardiomyopathy/ or takotsubo cardiomyopathy/ or arrhythmogenic cardiomyopathy/ or Chagas cardiomyopathy/ or ((dilated or idiopathic) adj1 cardiomyopath*).ti,ab. (117691) |
| 22 ventricular noncompaction/ (3043) |
| 23 brugada/ or Brugada syndrome/ (6963) |
| 24 Wolff-Parkinson-White/ (0) |
| 25 exp heart right ventricle dysplasia/ (6149) |
| 26 (arrhythmo* adj1 (ventric* or cardiomyo* or dysplas*)).ti,ab. (1683) |
| 27 ((malignant* or idiopathic) adj1 arrhythmia*).ti,ab. (1695) |
| 28 heart ventricle tachycardia/ or monomorphic ventricular tachycardia/ or heart arrhythmia/ or catecholaminergic polymorphic ventricular tachycardia/ or long QT syndrome/ or short QT syndrome/ or torsade des pointes/ or laminopathy/ (136578) |
| 29 cardiac sarcoidosis/ or heart amyloidosis/ or myocarditis/ or congenital heart disease/ (64278) |
| 30 or/7-29 (950074) |
| 31 6 and 30 (85703) |
| 32 therapy/ or treatment/ or management/ (434011) |
| 33 ((antiarrhythm* or antitachycard*) adj1 (agent* or drug* or medic* or pac*)).ti,ab. (12061) |
| 34 catheter ablation/ (34353) |
| 35 risk assessment/ or sudden death/pc [Prevention] (595758) |
| 36 genetic screening/ (90959) |
| 37 (cardiac adj1 (resynchroni?ation or defibrillat* or pacemaker*)).ti,ab. or implantable cardioverter defibrillator/ (57512) |
| 38 (CRT or CRTD or CRTP or ICD).mp. [mp=title, abstract, heading word, drug trade name, original title, device manufacturer, drug manufacturer, device trade name, keyword, floating subheading word, candidate term word] (132415) |
| 39 (ventric* adj1 (shock* or therap* or pac*)).ti,ab. (7715) |
| 40 treatment response/ or improve*/ or trend/ (283029) |
| 41 or/32-40 (1565739) |
| 42 31 and 41 (25958) |
| 43 (random$ or placebo$ or single blind$ or double blind$ or triple blind$).ti,ab. (1636160) |
| 44 exp cohort analysis/ (710518) |
| 45 exp longitudinal study/ (150064) |
| 46 exp prospective study/ (670844) |
| 47 exp follow up/ (1627405) |
| 48 cohort$.tw. (1112527) |
| 49 or/43-48 (4341893) |
| 50 (animal$ not human$).sh,hw. (2956484) |
| 51 exp review/ (2348633) |
| 52 (literature adj3 review$).ti,ab. (333731) |
| 53 (systematic$ adj2 (review$ or overview)).ti,ab. (276545) |
| 54 (meta?anal$ or meta anal$ or meta-anal$ or metaanal$ or metanal$).ti,ab. (262857) |
| 55 (book or conference paper or editorial or letter or review).pt. not exp randomized controlled trial/ (4313932) |
| 56 (random sampl$ or random digit$ or random effect$ or random survey or random regression).ti,ab. not exp randomized controlled trial/ (121189) |
| 57 or/50-56 (7676832) |
| 58 49 not 57 (3572534) |
| 59 42 and 58 (10579) |
| 60 limit 59 to yr="2015 -Current" (5066) |
| 61 limit 60 to english language (5010) |
| Database: Ovid MEDLINE(R) ALL <1946 to June 15, 2021> |
| Search Strategy: |
| 1 exp Death, Sudden, Cardiac/ (16219) |
| 2 (sudden adj2 death).mp. or sudden death/ (60060) |
| 3 cardiac death.mp. (26584) |
| 4 ((card* or sudden) adj1 (death or arrest)).ti,ab. (92136) |
| 5 (ventric* adj1 fibrillation).ti,ab. or heart ventricle fibrillation/ or "electrical storm (heart)"/ (18390) |
| 6 or/1-5 (125408) |
| 7 acute coronary syndrome/ or Myocardial Infarction/ or Anterior Wall Myocardial Infarction/ or Non-ST Elevated Myocardial Infarction/ or ST Elevation Myocardial Infarction/ or Inferior Wall Myocardial Infarction/ (188512) |
| 8 ST segment elevation/ (4929) |
| 9 infarction/ or ischemia/ or necrosis/ (115649) |
| 10 apoptosis/ and heart/ (1569) |
| 11 cell death/ and heart/ (173) |
| 12 heart failure/ (125034) |
| 13 congestive heart failure/ (125034) |
| 14 acute heart failure/ or congestive heart failure/ (125034) |
| 15 ischemic cardiomyopathy/ or alcoholic cardiomyopathy/ or nonischemic cardiomyopathy/ or restrictive cardiomyopathy/ or congestive cardiomyopathy/ or peripartum cardiomyopathy/ or hypertrophic cardiomyopathy/ or cardiomyopathy/ or takotsubo cardiomyopathy/ or arrhythmogenic cardiomyopathy/ or Chagas cardiomyopathy/ or ((dilated or idiopathic) adj1 cardiomyopath*).ti,ab. (71553) |
| 16 brugada/ or Brugada syndrome/ (3495) |
| 17 Wolff-Parkinson-White/ (5533) |
| 18 (arrhythmo* adj1 (ventric* or cardiomyo* or dysplas*)).ti,ab. (1048) |
| 19 ((malignant* or idiopathic) adj1 arrhythmia*).ti,ab. (1192) |
| 20 heart ventricle tachycardia/ or monomorphic ventricular tachycardia/ or heart arrhythmia/ or catecholaminergic polymorphic ventricular tachycardia/ or long QT syndrome/ or short QT syndrome/ or torsade des pointes/ or laminopathy/ (8011) |
| 21 cardiac sarcoidosis/ or heart amyloidosis/ or myocarditis/ or congenital heart disease/ (70005) |
| 22 or/7-21 (555299) |
| 23 6 and 22 (29542) |
| 24 therapy/ or treatment/ or management/ (8526) |
| 25 Therapeutics/ (8526) |
| 26 ((antiarrhythm* or antitachycard*) adj1 (agent* or drug* or medic* or pac*)).ti,ab. (13425) |
| 27 catheter ablation/ (33795) |
| 28 risk assessment/ or sudden death/pc [Prevention] (283352) |
| 29 genetic screening/ (40259) |
| 30 (cardiac adj1 (resynchroni?ation or defibrillat* or pacemaker*)).ti,ab. or implantable cardioverter defibrillator/ (27438) |
| 31 (CRT or CRTD or CRTP or ICD).mp. [mp=title, abstract, original title, name of substance word, subject heading word, floating sub-heading word, keyword heading word, organism supplementary concept word, protocol supplementary concept word, rare disease supplementary concept word, unique identifier, synonyms] (54918) |
| 32 (ventric* adj1 (shock* or therap* or pac*)).ti,ab. (6236) |
| 33 or/24-32 (442763) |
| 34 23 and 33 (6789) |
| 35 (random$ or placebo$ or single blind$ or double blind$ or triple blind$).ti,ab. (1328387) |
| 36 exp cohort analysis/ (2151131) |
| 37 exp longitudinal study/ (146290) |
| 38 exp prospective study/ (579226) |
| 39 cohort$.tw. (670082) |
| 40 or/35-39 (3550518) |
| 41 (animal$ not human$).sh,hw. (4798867) |
| 42 exp review/ (2821413) |
| 43 (literature adj3 review$).ti,ab. (317538) |
| 44 (systematic$ adj2 (review$ or overview)).ti,ab. (223666) |
| 45 (meta?anal$ or meta anal$ or meta-anal$ or metaanal$ or metanal$).ti,ab. (205125) |
| 46 (book or conference paper or editorial or letter or review).pt. not exp randomized controlled trial/ (4492402) |
| 47 (random sampl$ or random digit$ or random effect$ or random survey or random regression).ti,ab. not exp randomized controlled trial/ (100877) |
| 48 or/41-47 (9432949) |
| 49 40 not 48 (2981630) |
| 50 34 and 49 (2728) |
| 51 limit 50 to english language (2615) |
| 52 limit 51 to yr="2015 -Current" (1124) |

Table A3. Criteria for the development and evaluation of the ESC quality indicators for cardiovascular disease.

| Domain | Criteria |
| --- | --- |
| Importance | QI reflects a clinical area that is of high importance (e.g., common, major cause for morbidity, mortality, and/or health-related quality of life). |
|  | QI relates to an area where there is gap in care delivery and/or variation in practice. |
|  | QI implementation will lead to a meaningful improvement in patient outcomes. |
|  | QI may address under- and/or over-use of a test or treatment. |
| Evidence base | QI is derived from a clearly defined, acceptable evidence consistent with contemporary knowledge. |
|  | QI aligns with the respective ESC Clinical Practice Guideline recommendations. |
| Specification   | QI has clearly defined patient group to whom the measurement applies (denominator), including explicit eligibility criteria. |
|  | QI has clearly defined patient group for whom the QI is met (numerator), including explicit definition of QI meeting criteria. |
|  | QI has a minimum population level. |
| Validity | QI is able to correctly assess what it is intended to, adequately distinguishes between good- and poor-quality care, and compliance with the indicator would confer health benefits. |
| Reliability | QI is reproducible even when data is extracted by different people and estimates of performance on the basis of available data are likely to be reliable and unbiased. |
| Feasibility | QI may be identified and implemented with reasonable cost and effort |
|  | Data needed for the assessment is (or should be) readily available and easily extracted within an acceptable time frame. |
| Interpretability | QI is interpretable by healthcare providers, so that practitioners can understand the results of the assessment and take actions accordingly. |
| Actionability | QI is influential to the current practice where a large proportion of the determinants of adherence to the QI are under the control of healthcare providers being assessed. |
|  | This influence of QIs on behaviour will likely improve care delivery. |
|  | QI is unlikely to cause negative unintended consequences. |

ESC= European Society of Cardiology, QI=quality indicator

ACE= angiotensin converting enzymes; ARB=angiotensin receptor blocker; ARNI=angiotensin-receptor neprilysin inhibitor; eGFR= estimated glomerular filtration rate; MRA= Mineralocorticoid receptor antagonists; SGLT2=sodium-glucose transport protein 2.
